# The Atlanta Society of Medicine

**Published:** 1904-02

**Authors:** 


### SOCIETY REPORTS.

### THE ATLANTA SOCIETY OF MEDICINE.

The Atlanta Society of Medicine devoted its meeting of January 21 to a discussion of the X-rays in Medicine and Surgery. Remarks were made by the following gentlemen:

### THE RÖNTGEN RAYS.

*Dr. Wolff* said:—The history of electric discharges in rarefied air and gases goes back over 100 years before Crookes' communication to the *Chemical News* in 1879. In this communication Crookes refers to the experiments of Morgan in 1760, who noted phosphorescence in the exhausted receiver of an air-pump when an electric current had been discharged into it. Crookes revived the study of these phenomena. He worked with tubes of high vacuum and observed the disappearance of fluorescence when the vacuum became too high. He also observed a rectilinear radiation from the negative pole or cathode which he took to be particles of gas driven off with such high rate of speed as to heat the tube by impact against the walls or substances within it. This peculiar behavior of the gas he thought sufficient to distinguish it from that of all other gas or ordinary air and he called it the fourth or radiant state of matter. He also observed that the cathode rays were deflected by a magnet and intercepted by metallic substances. Before Crookes' experiments Geisler and Hittorf had also investigated electric discharges in vacuum tubes, the former noting the fact that the delicate glow or radiance which filled the tube could be deflected by a magnet. The perfection of the air-pump by Geisler and Bunsen paved the way to the vacuum-tube and greatly advanced its study under electric discharges.

For twenty years Crookes' work lay fallow when Hertz and his assistant, Lenard, observed that gold leaf and other thin sheets of metal introduced into the tube were penetrable by the cathode rays.

Lenard further noted that the same phenomenon occurred outside of the tube and that many opaque substances could be penetrated by the radiance; that certain other substances, as bario-platinum cyanide, fluoresced brilliantly when exposed to the rays, and that sensitized photographic plates were affected in the same manner as by ordinary light. These effects were presumed to be due to the cathode rays. It will be seen that these observers were very close to the discovery soon to follow.

Prof. William Conrad Röntgen of the University of Würzburg observed that paper coated with bario-platinum cyanide fluoresced even where the tube was enclosed in cardboard. This effect was noted to occur especially opposite the point in the tube of impingement of the cathode rays. It was not due to ordinary light, for the cardboard would occlude it. To use Röntgen's own words as contained in the opening of his address to the Physico-Medical Society of Würzburg, "If we pass a discharge from a large induction coil through a Hittorf's or a sufficiently exhausted Lenard's or Crookes' or a similar apparatus and cover the tube with a somewhat closely fitting mantle of black cardboard, we observe that a paper screen covered with crystals of barium platino-cyanide lights up brilliantly and fluoresces equally whether the treated side or the other is turned toward the discharge tube. It is easy to convince one's self that the cause of the fluorescence is the discharge from the tube." This was in December, 1895, and was the first succinct announcement of his discovery. A few weeks later Salvioni, of Perugia, described the same phenomena and devised the Cryptoscope.

Röntgen's investigations were so exhaustive that but little has since been added. The rays he denominated from their unknown value in the mathematical formula X-rays.

In a sufficiently exhausted tube wherever the cathode rays strike solid substances the X-rays are given off. The pear-shaped tube was then used, but has now given place to the focusing tube devised by Herbert Jackson, of King's College, London. In this and in numerous modifications the negative or cathode terminal is usually made of aluminum and is concave. Near it is placed the positive terminal or anti-cathode or anode which receives the discharge as a target at the narrowest part, and in this manner the rays originate from so small a surface that they can be focused within fairly narrow confines, permitting sharp shadows to be cast on the fluoroscopic screen or photographic plate.

The X-rays possess certain distinguishing peculiarities. They are rectilinear, incapable of refraction or defraction and to a very small extent of reflection. All bodies are penetrable by them in proportion to their atomic weight or density. They can discharge electrified bodies.

They cause a brilliant fluorescence in certain bodies as bario-platinum cyanide and tungstate of calcium, a fact upon which the fluoroscope is constructed.

They cast shadows of interposed substances upon sensitized photographic plates.

The question at this point very naturally arises, what is the nature of the X-rays? and a tentative answer involves a statement of the several theories which have been advanced. The corpuscular theory assumes that extremely small particles of matter are driven through the walls of the tube in front of the anode and thence between the atoms of substances in their tract.

Crookes leant to the view that the residual air in the tube was driven with great force through the tube from the cathode pole, the particles being converged by the dish shape of the cathode terminal, and struck the opposing plate of platinum at such high velocity as to raise the temperature.

Another hypothesis views the X-rays as longitudinal waves of such minuteness that no substance is sufficiently dense to reflect, refract or occlude them. All of these theories are more or less discredited, and consensus of opinion now leans to the electric nature of the X-rays. They are generated by an electric current, and their effect is electrical. According to this hypothesis, therefore, the X-rays are atoms separated into exceedingly small ions, each electrically charged, emerging from the cathode. These unite with the adjacent positive rays and penetrate, more or less, according to the electric resistance of the body against which they are directed.

Röntgen suggested the utility of the rays in surgery. In

May, 1896, Sydney Rowland wrote: "In bony diseases will the process be especially useful, for the reasons already given—that the bones stand out by presenting the greatest contrast to the rest of the tissues in the matter of transparency." This application was made without loss of time and the utilization of the Röntgen rays for diagnostic purposes in the detection of fractures, dislocations, the presence of foreign bodies, calculi and the like, is too well known to justify comment in this connection.

But not alone in surgery is their function as a diagnostic means called into service. They furnish very material aid in certain diseased conditions in the province of internal medicine by showing in contrast tissues of varying density, *e. g.*, tubercular consolidation of lung, pneumonia, pleurisy. But the great assistance offered by the X-rays in diagnostics does not exhaust their possibilities, for their utilization as a remedial measure exceeds their importance as a diagnostic agency.

It was early observed that dermatitis of varying degrees of severity, even extensive necrosis of tissue, followed prolonged exposure to the X-rays. This fact furnished a cue for the application of the rays in the treatment of malignant new formations, and the mass of evidence which has accumulated on this point supplies a complete justification and confirmation. Not only superficial cancerous processes are successfully treated by this means, but extensive deep-seated malignant growths, irremediable and unapproachable by other measures are often—too often to be mere coincidences—checked in their evolution and made to disappear by the beneficent power of the rays.

This fact is one of the greatest importance. The notable increase in cancer in the last half century and the unsatisfactory efforts of surgery to cope with the problem leave this disea-e one of the reproaches of medical science. Vaunted specifics exploited from time to time have one and all passed into the void. If solved at all, is the problem to be worked out through the medium of this or some other allied physical agent? When the results already achieved are considered while the agent is yet in its swaddling clothes, the promise and hope of the future seem to centre about it and point to it as the elect. There are various theories as to the rationale of the effects of the X-rays upon cancerous structures. The theory of cell inclusions of cancer being phases in the evolution of protozoa would lead to the conception that the rays were destructive of this low form of life and that healing was brought about by this parasiticidal power. But it is known that the rays do not possess this power to a marked degree, and the theory is further negatived by the circumstance that their capacity for destroying primitive life forms would be exhibited in all cases of cancer thus treated. The effect on cancer is not uniform, but shows many individual differences.

The cauterant effect scarcely explains the occurrences that take place in cancer under the influence of the rays. Deep-seated malignant growths are frequently profoundly influenced, while the superficial structures remain unaffected. If the effect were merely cauterant, as a matter of course the tissue would be destroyed in the order of propinquity to the source of the rays.

The alterative influence as increasing the reparative power of the normal tissue to the point where it is competent to cope with the abnormal is not enough to explain the effect, for it is not constant nor especially characteristic. The most plausible explanation of the mode of action of the rays is that offered by Skinner. The cancer-cell represents a reversion from normal to primitive type of tissue and the energizing of the former by the X-rays inhibits this tendency and checks growth, leaving to encapsulation of the pathologic tissue by envelopes of the normal structure. This eliminative act would produce atrophy or absorption of the aberrant tissue. The same process occurring rapidly would lead to a cutting off of the nutrition, jugulation and sloughing. This theory is fortified by a posteriori evidence.

Besides the application of the rays to the relief of cancer they occupy a field of great usefulness in dermatology and to some extent in general medicine.

In a brief outline sketch, such as this paper is designed to be, many important details as to the physics and therapy have necessarily been omitted. I have no doubt but that these will be fully considered in the discussion to follow.

From a limited but widening experience with radio-therapy, I

feel justified in the conclusion that in this we have a mechanism of inestimable value and it is rational to presume that another decade of assiduous investigation will place it upon a parity with the most efficient weapons which we now possess for combating disease.

REFERENCES: Transactions, Amer., Röntgen Ray Society, Vol. III., Pusey and Caldwell X-ray, Therapeutics of Journal of Advanced Therapeutics.

*Dr. M. B. Hutchins* expressed his appreciation of Dr. Wolff's paper giving the origin and development of the X-ray. He said that X-ray therapeutics was full of contradictions and the formulating of definite views as to the mode of action very difficult. As to apparatus, he could only speak definitely of the coil and electrolytic break as perfected by himself in his own machine—which did its work without trouble or variation. The vacuum of X-ray tubes is generally rather high, but can be regulated to some extent.

Whether the effect of X-rays is due to the light or to the electricity or to a combination of them is still a question. A recent theory attributes the light effect to the low vacuum tube and the electric to high vacuum. Dr. Hutchins believes it probable that we get both effects in the usual treatment.

He believed the manner of action in cancer, cases of which he had mostly in mind in the discussion, was best explained through the theory that the cancer cells were of lower vitality than the normal tissues, just as is the case with hair and nails, which are so easily influenced. Elaborating this idea somewhat he spoke of the more ready action of the X-ray upon cells with poor blood supply than upon those in vascular areas, the greater facility with which burns occurred in slightly vascular regions than on the tongue, for example. He believed the best effect in the treatment of cancer was to be had by pushing the treatment as rapidly as possible at first, or the half diseased tissues around are placed in a state of indolent inflammation or partial necrosis where the yet living disease cells find little resistance to their growth. A mild degree of "burn" is probably always helpful, a burn of more intensity often of great use. But should a great mass of semi—or fully, necrosed tissue be formed there is the grave danger of a toxemia, which may even prove fatal.

The X-ray is curative in the majority of cases of superficial cancer and a palliation, prolonging life, in many cases otherwise without hope of relief. In the treatment of many cases of skin disease it is the best remedy we have.

Regarding its effect in the relief of pain, its action is such, save where we so thoroughly break down the diseased cells, as we must, to check the disease, that we get a pain from the treatment to the patient indistinguishable from the old pain.

*Dr. R. R. Kime:*—One very important point in using the X-ray is the selection of cases, especially those suffering with malignant disease. The best interest of our patient requires discrimination and judgment on our part as to when we should use the X-ray or resort to surgery. To my mind this is a very important question. In early cases of malignancy deep-seated or beneath the cutaneous surface where it is possible to remove the diseased tissues, surgery is the remedy. In cases that are inoperable, that return after operation or in cases of cutaneous cancer or ulceration, then the X-ray should be used.

It is a serious matter to the patient to advise the X-ray in early malignant disease beneath the skin, thus causing delay in operative work, the electricity causing breaking down of the tumors or nodules thus by absorption favor dissemination and extension of the cancerous condition.

It is hardly probable that all the cancer cells are destroyed by the X-ray; if not, then absorption favors cell infiltration and invasion of other tissues. Again, the use of the X-ray may so affect the local tissues as to prevent the best results from operative work later. Our enthusiasm in the use of the X-ray should not cause us to overlook these important considerations.

*Dr. J. W. Duncan:*—I wish to congratulate those of you who have been experimenting with the X-rays on the success that you have had with same, and I insist that you do not become discouraged by not being able to understand the *modus operandi* of this remedy.

A few centuries since a potent remedy was discovered, its *modus operandi* was not known, neither is it well known at this time, and yet we all use it and will continue to use it. It came to stay. I

refer to mercury. I believe the X-ray is a potent remedy and that it has come to stay, and that it will continue to be used when we all shall have been buried.

*Dr. Cartledge:*—The gentleman here is about thirty-five years old, well nourished and with good family and personal history except as you see here.

This large tubercular ulcer began about two years ago as an apparent scratch of the skin just above the knee in front. It has been operated two or three times. Five months ago when I saw it, it occupied the entire front half of the leg from two inches above the patella up two-thirds the thigh. It was honeycombed with fistulous tracks through very hard, dead tissue. Arsenical paste with cocaine and orthoform were more painful than efficient.

Under ether, Drs. Giles Kelly and W. E. Johnson assisting, we cut away this large mass of lifeless tissue down nearly to the bone, and by accident into the knee synovial sac. The knee and bone did not seem involved. We were not able to cut quite to a healthy base and so we burnt the remaining suspicious flesh with pure carbolic acid, following with alcohol. We made the first dressing of moist two per cent. carbolic solution; our subsequent dressings were moist normal saline and the wound filled up and skinned over to a center less than the palm of the hand, when it suddenly became frosted over with tubercles which we fought down with pure carbolic acid, and the fight has been kept up practically till we began the X-ray twenty-one days ago. The changes noted from using X-ray are: the dead, horny flesh in center has become red and healthier in color, bleeds when cut and much sensation restored. Skinning over has not progressed any. Patient can use and bend leg much better and swelling over entire limb subsided. Static sparks likely accomplished some of the improvement in circulation.

X-ray exposures were begun with the X-ray tube about two inches from the ulcer for ten minutes alternate days; after a week, every day and increasing the exposure to the hard center five minutes each alternate day till yesterday (twentieth day) gave the center exposure fifty minutes with some amount of reaction as you see here by the bluish tumefaction. After four or five days' rest now, will resume exposure of five minutes to the entire surface and about twenty minutes to center.

### CASE NO. 2.

About three weeks ago, Dr. Brawner saw with me a cancer of face near nose on cheek about size of thumb-nail, which responded to the Ray in about two weeks. It had resisted every other effort and was running serum and a little blood constantly. Patient was about forty-eight years old; four months since her last period. Her uncle died of cancer. A sore of her breast around a receding nipple responded to local treatment according to Dr. Brawner's suggestion. When these two places were well, she told me of an offensive discharge from vagina. From the cervix I cut an elongated lip that looked bluish with two or three gray oozing patches; it was about one inch long and base occupied all lower lip of cervix. The very offensive discharge cleared after four or five X-ray treatments, and now (three weeks) the ulcer is perfectly healed, but purple tumefaction there still. Examination by Dr. Chas. Andrews showed it to be epithelioma.

*Dr. Gaston* said there were many conflicting points in the evidence obtained from X-ray photographs. He was more willing to accede to a good skiagraph than a fluoroscope view of a bone or other portion of the body. His experience with a bullet lodged in a boy's back showed the illusions of the skiagraphs as the bullet could not be found where it seemed to be in several pictures. The methods of localization should be adopted to ascertain this.

In the case of Dr. Cartledge which Dr. Gaston had seen, there was also conflicting evidence. The skiagraph showed a dense body near the femur, and then a longer piece longitudinally as though in the flesh. The fluoroscope does not confirm this, but the general appearance of the external soft parts is that which often attends caries of the bone. Hence another skiagraph will be taken to determine the difference.

